# Bradycardia in the setting of postpartum preeclampsia and influenza A: A case report

**DOI:** 10.1016/j.crwh.2026.e00785

**Published:** 2026-01-15

**Authors:** Dalia Rahmon, Kelly Dubay, Sarah Deighton-Collins

**Affiliations:** Henry Ford Providence, Department of Obstetrics and Gynecology, 16001 W Nine Mile Rd, Southfield, MI 48075, United States

**Keywords:** Postpartum, Bradycardia, Preeclampsia, Influenza, Magnesium Sulfate

## Abstract

Postpartum bradycardia is a rare clinical finding, with limited guidance regarding its evaluation and management. Prior literature suggests that postpartum bradycardia is most commonly associated with preeclampsia, underlying cardiac disease, medication effects, or neuraxial anesthesia. In patients with preeclampsia, management may be particularly challenging, as magnesium sulfate—used for seizure prophylaxis—is theorized to exacerbate bradycardia.

This report concerns the case of a 33-year-old woman (G3P3003) with an otherwise uncomplicated pregnancy and cesarean delivery who was diagnosed with Influenza A on postpartum day 1 and treated with oseltamivir. She was readmitted on postpartum day 7 with epigastric pain and shortness of breath and was found to have severe sinus bradycardia (heart rate 32–42 beats per minute) and highly elevated blood pressure, consistent with new-onset postpartum preeclampsia with severe features. Cardiac evaluation, including electrocardiography and transthoracic echocardiography, revealed no structural or ischemic abnormalities. The patient was treated with magnesium sulfate for seizure prophylaxis and antihypertensive therapy. Despite persistent bradycardia, she remained hemodynamically stable, and her heart rate gradually normalized over three days without additional intervention. She was discharged in stable condition and remained asymptomatic at follow-up.

This case highlights postpartum bradycardia as a potential presenting sign of delayed-onset preeclampsia, even following a normotensive pregnancy. Although magnesium sulfate and recent influenza infection were considered as contributing factors, the clinical course supported preeclampsia as the primary etiology. Postpartum bradycardia is often benign and self-limited; however, thorough evaluation is essential to exclude cardiac pathology and guide appropriate management.

## Highlights

• Preeclampsia-associated bradycardia presents diagnostic challenges and requires an individualized management approach
• Medication history should be reviewed as a possible cause of bradycardia.
• Isolated bradycardia is often benign and self-resolving.
• Cardiac evaluation is recommended for patients with significant postpartum bradycardia.
• More research is needed to guide evidence-based management of postpartum bradycardia.

## 1 Introduction

Pregnancy and the postpartum period are associated with significant physiologic cardiovascular changes. Compared with pregnancy, the postpartum state is characterized by decreases in cardiac output, heart rate, and circulating blood volume as the body returns to its pre-pregnancy baseline [1]. Postpartum bradycardia is extremely rare, and current knowledge is limited to a small number of case reports and literature reviews. There are no established guidelines for its management, which can be particularly challenging in the setting of preeclampsia.

This report describes a patient with an otherwise uncomplicated pregnancy and delivery who was diagnosed with influenza on postpartum day 1 and subsequently readmitted on postpartum day 7 with severe bradycardia in the setting of new-onset postpartum preeclampsia with severe features.

## 2 Case Presentation

A previously healthy 33-year-old woman (G3P3003) presented to the emergency department on postpartum day 7 with epigastric pain and shortness of breath. She had undergone a scheduled repeat cesarean delivery following an uncomplicated pregnancy. Her immediate postpartum course was notable for Influenza A, diagnosed on postpartum day 1, for which she was treated with a five-day course of oseltamivir. She was discharged home in stable condition on postpartum day 2.

On presentation to the emergency department, her blood pressure was labile, ranging from 98/68 to 168/106 mmHg. Her pulse was 32–42 beats per minute (bpm), oxygen saturation 96–100% on room air, and temperature 97.3 °F (36.3 °C). Physical examination revealed reproducible epigastric tenderness, 2+ deep tendon reflexes, and a bradycardic pulse. Laboratory evaluation demonstrated anemia with a hemoglobin level of 10.2 g/dL (reference range 12.0–16.0 g/dL) but was otherwise unremarkable. Comprehensive metabolic panel, lipase, and thyroid-stimulating hormone levels were within normal limits. Troponin T was 10 ng/L (reference range 0–10 ng/L), and pro–B-type natriuretic peptide was elevated at 532 pg/mL (reference range 50–177 pg/mL).

Electrocardiography (EKG) demonstrated sinus bradycardia without ST-T segment abnormalities. Chest radiography and computed tomography angiography of the chest revealed findings consistent with atypical pneumonia and small dependent pleural effusions. Transthoracic echocardiography (TTE) demonstrated a left ventricular ejection fraction of 50–55% with no evidence of structural heart disease or significant myocardial dysfunction.

The patient met diagnostic criteria for preeclampsia with severe features due to persistent severe-range blood pressures (>160/110 mmHg) more than four hours apart. She was started on intravenous magnesium sulfate for seizure prophylaxis (4 g loading dose followed by 2 g/h maintenance infusion). Intravenous hydralazine 10 mg was administered as needed for sustained severe-range blood pressures. Long-acting antihypertensive therapy was deferred due to the labile nature of her blood pressures. Serum magnesium levels increased appropriately from 1.5 mEq/L to a therapeutic level of 4.4 mEq/L. She was also treated with ceftriaxone and azithromycin for pneumonia.

Despite therapy, the patient continued to exhibit significant bradycardia, in the range of 30–40 bpm. Magnesium sulfate was discontinued after approximately 12 h of therapy, and she was monitored on continuous telemetry. Over the next 48 h, she continued to experience intermittent severe-range blood pressures (≥160/110 mmHg), requiring additional intravenous hydralazine. She was ultimately started on procardia 30 mg daily to optimize blood pressure control.

Her heart rate gradually improved from a nadir of 32 bpm to 77 bpm by hospital day 3 without further intervention. She was discharged home on nifedipine, doxycycline, and prophylactic enoxaparin. At follow-up two weeks later, she remained asymptomatic with normal vital signs (blood pressure 112/74 mmHg, heart rate 99 bpm).

## 3 Discussion

Postpartum bradycardia is a rare clinical phenomenon. In a cohort study of over 1000 postpartum women, the median (3rd-97th centile) heart rate was 84 (59–110) bpm on the day of delivery, decreasing to 75 (55–101) bpm by 14 days postpartum [2]. Another study found that postpartum bradycardia was most commonly associated with preeclampsia, with other etiologies including underlying cardiac conditions, medications, and neuraxial anesthesia [3].

Multiple case reports have documented bradycardia in the setting of elevated blood pressure, suggesting it may serve as a premonitory sign of hypertensive disorders of pregnancy [4], [5], [6], [7]. In patients with preeclampsia, bradycardia has been reported to occur at a median of four days postpartum, corresponding with the typical postpartum rise in blood pressure [3]. This pattern was observed in the present patient following a normotensive pregnancy. Although the underlying pathophysiology remains unclear, proposed mechanisms include increased intracranial pressure with activation of the Cushing reflex, posterior reversible encephalopathy syndrome, and increased vagal tone mediated by arterial and cardiac baroreceptors [3], [8].

While bradycardia has been reported during magnesium sulfate therapy, it is not a recognized complication at therapeutic levels in patients without underlying cardiac disease [4], [9], [10]. In the present case, magnesium sulfate was discontinued after 12 h due to persistent bradycardia; however, the patient subsequently required additional antihypertensive therapy for severe-range blood pressures. This raises the clinical question of whether magnesium sulfate should be continued in patients with significant bradycardia when serum levels remain therapeutic and no alternative etiology is identified.

Although a cardiac evaluation is warranted in patients with postpartum bradycardia, it is important to recognize that this condition is often benign, self-limited, rarely requiring invasive intervention [3]. In the present patient, evaluation included cardiac biomarkers, EKG, chest imaging, and TTE, all of which were reassuring aside from an elevated pro-BNP. CT angiography of the chest may be considered in cases with atypical presentations where pulmonary embolism remains a concern.

A thorough medication history is essential when evaluating postpartum bradycardia. In the present case, the patient had completed a course of oseltamivir one day prior to presentation. Both influenza infection and oseltamivir use have been associated with bradycardia or relative pulse reduction in up to 43% of patients [11], [12], [13]. Oseltamivir-associated bradycardia typically occurs within two days of initiation and resolves within 12 h of discontinuation [12], [13]. In contrast, this patient's bradycardia persisted for several days after completing therapy, supporting preeclampsia as the primary etiology.

Following discharge, routine postpartum follow-up with in-office blood pressure monitoring is recommended. Patients should be counseled regarding their history of delayed-onset postpartum preeclampsia and the associated risk of recurrence in future pregnancies. Early prenatal care, close blood pressure surveillance during pregnancy and the postpartum period, and prompt evaluation of symptoms suggestive of hypertensive disorders are essential. Preventive strategies, including the use of low-dose aspirin in future pregnancies per current obstetric guidelines, should be discussed. When antihypertensive therapy is required, medications with negative chronotropic effects may best be avoided in patients with a history of significant bradycardia. Importantly, postpartum bradycardia is typically benign and self-resolving and does not independently contraindicate future pregnancies.

## 4 Conclusion

Postpartum bradycardia is a rare but clinically significant condition that presents unique diagnostic and management challenges. This case highlights the complexities of managing bradycardia in the setting of preeclampsia with severe features, emphasizing the need for individualized treatment approaches. While the etiology of postpartum bradycardia is unclear, it is important to consider all contributing factors, including medication history. A cardiac workup should be performed on all patients with significant bradycardia; however, it is important to note that this condition is often benign and self-limited. Further research is needed to clarify the underlying mechanisms and to establish evidence-based guidelines for the management of postpartum bradycardia.

## Contributors

Dalia Rahmon contributed to patient care, conception of the case report, acquiring and interpreting the data, drafting the manuscript, undertaking the literature review and revising the article critically for important intellectual content.

Kelly Dubay contributed to patient care, conception of the case report, acquiring and interpreting the data, drafting the manuscript, undertaking the literature review and revising the article critically for important intellectual content.

Sarah Deighton-Collins contributed to patient care and revising the article critically for important intellectual content.

All authors approved the final submitted manuscript.

## Patient consent

Written informed consent was obtained from the patient for their anonymized information to be published in this article.

## Provenance and peer review

This article was not commissioned and was peer reviewed.

## Funding

No funding from an external source supported the publication of this case report.

## Declaration of competing interest

The authors declare that they have no competing interest regarding the publication of this case report.
